# Morbidity matters: challenges for research

**DOI:** 10.3399/bjgp15X684301

**Published:** 2015-03-30

**Authors:** FD Richard Hobbs, Maureen Baker, Dame Sally C Davies

**Affiliations:** NIHR School for Primary Care Research, University of Oxford, Oxford.; Royal College of General Practitioners, London.; Office of the Chief Medical Officer for England, Department of Health, London.

Multimorbidity is an immediate and expanding challenge for many health systems, including the NHS. It can be defined as the coexistence of several chronic diseases or medical conditions in one person. It is increasingly common among older people, as effective interventions reduce fatal events but increase the prevalent disease population, and life expectancy of the general population continues to rise. As a result, multimorbidity is now a fundamental care issue that presents a number of complex challenges for health systems, patients, and clinicians to address.

Almost one-in-three people in the UK — or 15 million individuals — have a long-term condition (LTC). Half the population aged >60 years now has a LTC. Those with LTCs account for half of all GP appointments and 70% of inpatient bed days. It is estimated that treatment and care of these patients account for 70% of the acute care budget in England; over two-thirds of NHS expenditure for one-third of the population. Those with LTCs are also likely to have a lower quality of life.^1^

On Friday 7 November 2014, the National Institute for Health Research (NIHR) and Royal College of General Practitioners (RCGP) jointly hosted the Multimorbidity Research Workshop at the RCGP, attended by more than 50 leading experts in the field of complex LTCs. The aims of the workshop were to identify challenges to research on multimorbidity and to prioritise research questions for research funders that may improve patient experience and management in primary and hospital care.

## DRIVING THE DEBATE

Professor Chris Salisbury, chair of the Scientific Foundation Board of the RCGP, set the scene with a presentation on ‘Challenges for Research: What are they and how can they be addressed?’ Although GPs, as expert medical generalists, are more likely to take a holistic and comprehensive view of patient care, even in general practice patients are invited to clinics for individual conditions, or clusters of conditions, and delivered structured care for that specific disease; for example, diabetes. However, many people do not have one LTC but several conditions, either concordant (linked problems, often with similar interventions advocated), discordant (unrelated problems), or both. Patients with multimorbidity therefore find themselves attending multiple distinct clinics, which may be inconvenient and inefficient for themselves and for practices.^2^ They may receive conflicting advise and/or prescribed drugs from different practitioners, and may also feel that the things that matter most to them — the problems that directly affect their quality of life — are not necessarily the highest priority for each doctor or nurse consulted.

Existing systems that address circumscribed conditions were never designed to manage the levels of multimorbidity now present in 50% of patients aged ≥75 years and increasingly prevalent within deprived communities.^3^ As a result, there is currently poor coordination in primary care and the interface with secondary care. Further, since guidelines recommend explicit management targets for specific conditions, care is often determined by individual target metrics, or a ‘tick-box’ application of guidelines that sustains a disease-by-disease approach to treating patients, rather than necessarily prioritising treatments that most directly affect their quality of life.

## CHALLENGES FACED BY PATIENTS

An early priority in addressing the challenges facing patients with multimorbidity is to discover what those patients actually want, and how they would like to see their care improved. Repeated suggestions during the workshop included: comprehensive reviews of patients as a whole rather than for each condition; identifying patient priorities such as pain relief and mobility as early as possible; investigating whether fewer but longer consultations help; exploring ways to simplify complex drug regimens; delivering emotional and psychological support to ensure the mental wellbeing of patients with physical health problems; and providing fresh assessment and robust reporting of the impact of any new approaches guided by patient feedback as well as outcomes.

Gopa Mitra, Chair of the Patient Partnership Group, RCGP, used vivid current experiences of patients with multimorbidity to provoke answers to the question: ‘What is it like to be a patient with multimorbidities?’. The prevailing message throughout her presentation emphasised that patients are more than the sum of their individual diseases, some want to be involved more in their own care plans, and that some desire more options for self-management. An example of one general practice posting a sign that reads ‘One appointment for one problem please’ encapsulated the challenge facing both patients and clinicians.

Most delegates agreed that patient-centred care approaches offered a number of benefits applicable to multimorbidity. The drivers behind the NHS House of Care model^4^ — a need to grasp the scale of the problem, rethink the way we deal with LTCs, and shift to a model that focuses predominantly on more patient determined outcomes — were well received. It was considered important that new studies addressed the degree to which these approaches actually improved patient outcomes.

Considering services from a patient’s point of view offers a way of maintaining perspective and understanding the need for improvements in clinical engagement. Despite the differing challenges that diverse subsets of comorbidities in individual patients present, a holistic approach to patient care planning and support for self-management of conditions by patients still remain well-supported concepts with clinicians.

## COMPLEXITIES OF CARE

In the final presentation Carl May, Professor of Healthcare Innovation, University of Southampton, asked: ‘How do we develop new interventions to promote the provision of high-quality care that might meet the needs of people with multimorbidity?’. The question was prompted by consideration of the burden of treatment.^5^ Clinicians persevere with legacy systems developed to deal with a single-condition focus of acute illness and infection rather than the modern ubiquity of multimorbidity, which may not fit the traditional approaches of clinical practice. The result is fragmented care for patients with multimorbidity, which may, perversely, increase both the burden of symptoms and the burden of treatment for patients and extended social networks.

## ALL IN A DAY’S WORKSHOP

An important element of the day saw attendees split into prioritisation workshops to consider topics for future research commissioning. Each group identified research questions that could improve patient and public outcomes, as well as NHS services, conceptualising research questions in a ‘PICO’ style format addressing Population, Intervention, Comparator, and Outcomes.

The first workshop group was tasked with generating new evidence to transform the effectiveness of interventions and improve outcomes in older patients with multimorbidity. Previous interventions have had limited success in improving drug prescriptions and adherence, and therefore mixed effects in improving patient health. The group prioritised the synthesis of evidence for the ‘real world’, understanding that there was no ‘magic bullet’ solution to such a complex paradigm with correspondingly high numbers of comorbidity sub-groups. However, delegates were also keen to reject any notion that the problem was too broad to address with fresh research, and asserted that overcoming the complex taxonomy of multimorbidity was an important challenge in itself. The group identified value in re-analysing existing work into various sub-populations of comorbidity to explore for possible sub-group benefits missed by a primary focus on mean response, with attention to outcomes for both concordant and discordant conditions.

The question posed to the second group was ‘How can we use existing evidence and data?’. Although many patients with multimorbidity experience poorer health outcomes than those with single chronic diseases, some research is already being conducted in this area. The limitation of trials that may have excluded patients with comorbidity and multimorbidity was raised, as well as the potential value of routine observational data and social policy data. The group was sensitive to the demands placed on front-line care, but considered that experimentation was warranted on the length and focus of patient appointments. Examples of existing holistic care approaches were discussed, which are candidates for fuller evaluations.

The third group discussed patient engagement and participation. Older people with multimorbidities are frequently excluded from randomised controlled trials that evaluate single diseases, limiting treatment data. The group identified an evidence gap, with only a small number of qualitative studies related to multimorbidity. There is also potential for evaluation of interventions for improved patient engagement (for example, technology, self-management, triangulation of care, and reduced burden on patients) and barriers to enhanced participation (for example, social inequality, low-levels of literacy, and poor links between health care and social care). Tractable research topics suggested included exploring the cost of self-management for patients, how to enable clinicians to reduce the treatment burden for patients, and the assessment of infrastructure and care for male patients.

The final workshop group was concerned with implementation and informing practice in the care of patients with multimorbidity. They particularly considered the impact of mental health problems in patients with physical LTCs, focusing on identifying barriers to informing and implementing new approaches to multimorbidity, highlighting issues such as capacity, time, and cost of service delivery change; backfill requirements of longer consultations; a lack of time to judge the results of any changes; and a lack of understanding of many comorbidity or polypharmacy combinations. Potential research focus ranged from ‘How do we elicit change within primary care?’ and ‘How can non-medical interventions be introduced more widely within translational research?’ to ‘How do we demonstrate how multimorbidity research addresses areas of greatest need (social and health), and assists workforce capacity?’ and, ‘How can multimorbidity research deliver products which will help with adoption and diffusion for professionals?’

## NEXT STEPS

This RCGP/NIHR workshop summarised the importance of multimorbidity, highlighting the comparative lack of evidence, beyond descriptive surveys, to guide clinical practice. These conclusions provide the rationale for the forthcoming NIHR-themed research call on multimorbidity in older people, which was launched on 27 January 2015. It is a timely and important call.
